# 3D Brain Segmentation Using Dual-Front Active Contours with Optional User Interaction

**DOI:** 10.1155/IJBI/2006/53186

**Published:** 2006-10-12

**Authors:** Hua Li, Anthony Yezzi, Laurent D. Cohen

**Affiliations:** ^1^ School of Electrical and Computer Engineering, Georgia Institute of Technology, Atlanta, GA 30332, USA; ^2^ CEREMADE, CNRS UMR 7534, Université Paris-Dauphine, Paris Cedex 75775, France

## Abstract

Important attributes of 3D brain cortex segmentation algorithms include robustness, accuracy, computational efficiency, and facilitation of user interaction, yet few algorithms incorporate all of these traits. Manual segmentation is highly accurate but tedious and laborious. Most automatic techniques, while less demanding on the user, are much less accurate. It would be useful to employ a fast automatic segmentation procedure to do most of the work but still allow an expert user to interactively guide the segmentation to ensure an accurate final result. We propose a novel 3D brain cortex segmentation procedure utilizing dual-front active contours which minimize image-based energies in a manner that yields flexibly global minimizers based on active regions. Region-based information and boundary-based information may be combined flexibly in the evolution potentials for accurate segmentation results. The resulting scheme is not only more robust but much faster and allows the user to guide the final segmentation through simple mouse clicks which add extra seed points. Due to the flexibly global nature of the dual-front evolution model, single mouse clicks yield corrections to the segmentation that extend far beyond their initial locations, thus minimizing the user effort. Results on 15 simulated and 20 real 3D brain images demonstrate the robustness, accuracy, and speed of our scheme compared with other methods.


Hindawi Publishing Corporation
International Journal of Biomedical Imaging
Volume 2006, Article ID 53186, Pages 1–17
DOI 10.1155/IJBI/2006/53186

### 3D Brain Segmentation Using Dual-Front Active Contours with Optional User Interaction

**Hua Li,[1] Anthony Yezzi,[1] and Laurent D. Cohen[2]**

[1] *School of Electrical and Computer Engineering, Georgia Institute of Technology, Atlanta, GA 30332, USA*
[2] *CEREMADE, CNRS UMR 7534, Université Paris-Dauphine, 75775 Paris Cedex, France*

Received 1 December 2005; Revised 30 May 2006; Accepted 31 May 2006

Important attributes of 3D brain cortex segmentation algorithms include robustness, accuracy, computational efficiency, and facilitation of user interaction, yet few algorithms incorporate all of these traits. Manual segmentation is highly accurate but tedious and laborious. Most automatic techniques, while less demanding on the user, are much less accurate. It would be useful to employ a fast automatic segmentation procedure to do most of the work but still allow an expert user to interactively guide the segmentation to ensure an accurate final result. We propose a novel 3D brain cortex segmentation procedure utilizing dual-front active contours which minimize image-based energies in a manner that yields flexibly global minimizers based on active regions. Region-based information and boundary-based information may be combined flexibly in the evolution potentials for accurate segmentation results. The resulting scheme is not only more robust but much faster and allows the user to guide the final segmentation through simple mouse clicks which add extra seed points. Due to the flexibly global nature of the dual-front evolution model, single mouse clicks yield corrections to the segmentation that extend far beyond their initial locations, thus minimizing the user effort. Results on 15 simulated and 20 real 3D brain images demonstrate the robustness, accuracy, and speed of our scheme compared with other methods.

Copyright © 2006 Hua Li et al. This is an open access article distributed under the Creative Commons Attribution License, which permits unrestricted use, distribution, and reproduction in any medium, provided the original work is properly cited.

### 1. INTRODUCTION

Three-dimensional image segmentation is an important problem in medical image analysis. Determining the location of the cortical surface of the human brain from MRI imagery is often the first step in brain visualization and analysis. Generally, the normal human brain consists of three kinds of tissues: white matter (WM), gray matter (GM), and cerebrospinal fluid (CSF). Due to the geometric complexity of the human brain cortex, manual slice by slice segmentation is quite difficult and time consuming. Thus, many semiautomatic or automatic segmentation methods have been proposed in recent years.

The active contour model, which was first introduced in [1] as "snakes," is an energy minimization method and has been widely applied in medical imaging. Cohen first extended snakes to 3D models and also used them for 3D medical image segmentation [2–4]. Malladi et al. [5] also showed their application to 3D medical image segmentation. Afterwards, they proposed a hybrid strategy of level set/fast marching segmentation for 3D brain cortex segmentation [6]. In their method, a small front is initialized inside the desired region, and then the fast marching method [7] is used to greatly accelerate the initial propagation from the seed structure to the near boundary, which gives a fast and rough initialization to a costly segmentation. Then the narrow band level set method [8] is used to achieve the final result.

In addition to the above method, numerous contributions [4, 9–19] have been made on the segmentation of complex brain cortical surfaces based on active contour models. Davatzikos and Bryan [9] used the homogeneity of intensity levels within the gray matter region to introduce a force that would drive a deformable surface towards the center of the gray matter, and built the cortex representation by growing out from the white matter boundary. Based on this parameterization, the cortical structure is characterized through its depth map and curvature map. This model explicitly used the structural information of the cortex. However, close initialization and significant human interaction are needed to force the ribbon into sulcal folds.

Pham and Prince proposed an adaptive fuzzy segmentation method [15] for brain cortex extraction from images which have been corrupted by intensity inhomogeneities. In their method, the minimized objective function has two additional regularization terms added to the gain field, which is different from object functions in standard fuzzy $C$-means algorithms [20]. Their method iteratively estimates the fuzzy membership functions for each tissue class, the mean intensities of each class, and the inhomogeneity of the processed image, and models the intensity inhomogeneities to a smoothing varying gain field. They reported that their method yields lower error rates than standard fuzzy $C$-means algorithms [20].

Lately, Xu et al. [13] described a systematic method for obtaining a surface representation of the geometric central layer of the brain cortex. It is a four-step method including brain extraction, fuzzy segmentation, isosurface generation, and a deformable surface model using gradient vector flow [21]. They defined the central cortical layer as the layer lying in the geometric center of the cortex, and applied a deformable surface model on the membership functions computed by the adaptive fuzzy segmentation [15] instead of image intensity volumes. Han et al. [18] also proposed a topology-preserving geometric deformable surface model for brain segmentation.

Teo et al. [11] proposed a four-step segmentation method based on deformable models. They first segmented white matter and cerebral spinal fluid regions by anisotropic smoothing of the posterior probabilities of different predefined regions, then selected the desired white matter components and verified and corrected the white matter structure based on cavities and handles. Finally a representation of the gray matter was created by constrained growth starting from the white matter boundary. Their work focused on creating a representation of cortical gray matter for functional MRI visualization. Dale et al. [12] concentrated on cortical surface-based analysis. They started by deforming a tessellated ellipsoidal template into the shape of the inner surface of the skull under the influence of MRI-based forces and curvature reducing forces. White matter was then labeled and the cortical surfaces were reconstructed with validation of topology and geometry.

MacDonald et al. [16] proposed to use an intersurface proximity constraint in a two-surface model of the inner and outer cortex boundaries in order to guarantee that surfaces do not intersect themselves or each other. Their method was an iterative algorithm for simultaneous deformation of multiple surfaces formulated as an energy minimization problem with constraints. This method was applied to 3D MR brain data to extract surface models for the skull and the cortical surfaces, and it took advantage of the information of the interrelation between the surfaces of interest. However, its main drawbacks include an extremely high computational expense and the difficulty of tuning the weighting factors of the cost function, due to the complexity of the problem.

Zeng et al. [14] used the fact that each cortical layer has a nearly constant thickness to design a coupled surfaces model, in which two embedded surfaces evolve simultaneously, each driven by its own image-based forces so long as the intersurface distance remained within a predefined range. They measured the likelihood of a voxel to be on the boundary between two issues and used this as a local feature to guide the surface evolution. Gomes and Faugeras [22] also implemented the above coupled surfaces model with a different scheme that preserves the level-set surface representation function as a distance map, so that reinitialization is not required every iteration. Goldenberg et al. [17] proposed a similar coupled surfaces principle and developed a model using a variational geometric framework. In their method, surface propagation equations are derived from minimization problems and implemented based on a fast geodesic active contours approach [23] for improving computational speed.

Kapur et al. [10] presented a method for the segmentation of brain tissues from MRI images which is a combination of EM segmentation, binary mathematical morphology, and active contours. EM segmentation is used for intensity-based correction and data classification. Binary morphology and connectivity is used for incorporation of topological information, and balloon-based deformable contours [3] are used for the addition of spatial information to the segmentation process. Cristerna et al. [19] proposed a hybrid methodology for brain multispectral MRI segmentation, which couples a Bayesian classifier based on a radial basis network with an active contour model based on cubic spline interpolation.

Many other automatic methods for brain cortex segmentation using T1-weighted or multispectral MR data were also proposed such as histogram threshold determinations [24, 25], fuzzy set methods [15, 26], Bayesian methods [27], Markov random field methods [28–31], expectation-maximization (EM) algorithms [10, 32], and so on.

These methods were aimed at segmenting the brain tissues automatically, and eliminating or nearly eliminating user interaction for choosing the parameters of the automatic process, setting initial surfaces for surface evolution, or restricting regions to be processed. However, there is something to be said for allowing trained users to guide the segmentation process with their expert knowledge of what constitutes a correct segmentation. Methods that allow simple and intuitive user interaction (while minimizing the need for such interaction as much as possible) are therefore potentially more useful than totally automatic methods given the importance of high accuracy and detail in cortical segmentation.

In this paper, we propose a novel 3D brain cortex segmentation scheme based on dual-front active contours which are faster and yield flexibly global image-based energy minimizers related to active regions compared to other active contours models. This scheme also adapts easily to user interaction, making it very convenient for experts to guide the segmentation process by adding useful seed points with simple mouse clicks. This scheme is very fast and the total computational time is less than 20 seconds. Experimental results on 15 simulated and 20 real 3D brain images demonstrate the robustness of the result, the high reconstruction accuracy, and the low computational cost compared with other methods.

This paper is organized into the following sections. In Section 2, we review the dual front active contour model and a number of its properties. In Section 3, we extend dual-front active contours to 3D brain cortex segmentation. Section 3.1 introduces the overall diagram of 3D brain cortex segmentation based on dual-front active contours. Section 3.2 describes how to choose active regions and potentials for the dual-front active contours based on histogram analysis. In Section 4, we show experimental results on various simulated and real brain images as well as comparisons with other cortex segmentation methods. We also demonstrate some of the features and properties of our scheme such as simple and useful user interactions and high computational efficiency. Finally, conclusions and future research work are presented in Section 5.

### 2. DUAL-FRONT ACTIVE CONTOURS

The basic idea of dual-front active contours was proposed in [33, 34] for detecting object boundaries. It is an iterative process motivated by the minimal path technique [35] utilizing fast sweeping methods [36, 37]. In this section, we give a review of dual-front active contours.

### 2.1. Background-minimal path techniques

Since dual-front active contours are motivated by the minimal path technique proposed by Cohen and Kimmel [35, 38, 39], we give a brief summary of this technique in this subsection. Their technique is a boundary extraction approach which detects the global minimum of an active contour model's energy between two user-defined points located on the boundary, and avoids local minima arising from the sensitivity to initialization in geodesic active contours [4, 40]. Contrary to energy functionals defined in snakes [1], they proposed a simplified energy minimization model,

$$E(C) = \int_{\Omega} \{w + P(C(s))\} ds = \int_{\Omega} \widetilde{P}(C) ds, \tag{1}$$

where $s$ represents the arc-length parameter of a curve $C(s) \in \mathbb{R}^n$. $P$ is a pointwise potential associated to image features, while $w$ is a real positive constant.

Given a potential $P > 0$ that takes lower values near the desired boundary, the objective of the minimal path technique is to look for a path (connecting two user-defined points) along which the integral of $\widetilde{P} = P + w$ is minimal. In [35], a minimal action map $U_{p_0}(p)$ was defined as the minimal energy integrated along a path between a starting point $p_0$ and any point $p$:

$$U_{p_0}(p) = \inf_{A_{p_0,p}} \left\{ \int_{\Omega} \widetilde{P}(C(s)) ds \right\} = \inf_{A_{p_0,p}} \{E(C)\}, \tag{2}$$

where $A_{p_0,p}$ is defined as the set of all paths between $p_0$ and $p$. The value of each point $p$ in the minimal action map $U_{p_0}(p)$ corresponds to the minimal energy integrated along a path starting from point $p_0$ to point $p$.

So the minimal path between point $p_0$ and point $p$ can be easily deduced by calculating the action map $U_{p_0}(p)$ and then sliding back from point $p$ to point $p_0$ via gradient descent.

They also noted that given a minimal action map $U_{p_0}$ to point $p_0$ and a minimal action map $U_{p_1}$ to point $p_1$, the minimal path between points $p_0$ and $p_1$ is exactly the set of points $p_g$ which satisfy

$$U_{p_0}(p_g) + U_{p_1}(p_g) = \inf_{p} \{U_{p_0}(p) + U_{p_1}(p)\}. \tag{3}$$

A saddle point $p'$ is the first point where two action maps $U_{p_0}$ and $U_{p_1}$ meet each other, which means that $p'$ satisfies $U_{p_0}(p') = U_{p_1}(p')$ and (3) simultaneously. The minimal path between points $p_0$ and $p_1$ may also be determined by calculating $U_{p_0}$ and $U_{p_1}$ and then, respectively, sliding back from the saddle point $p'$ on the action map $U_{p_0}$ to point $p_0$ and from the saddle point $p'$ on the action map $U_{p_1}$ to point $p_1$ according to the gradient descent. This idea was used in [39] for finding closed contours as a set of minimal paths from an unstructured set of points. It was also used in [41] in order to reduce the computational cost of a variety of fast marching applications. In order to compute $U_{p_0}(p)$, they formulated a PDE equation:

$$\frac{\partial L(v,t)}{\partial t} = \frac{1}{\widetilde{P}} \vec{n}(v,t), \tag{4}$$

to describe the level sets $L$ of $U_{p_0}$, where "time" $t$ represents heights of the level sets of $U_{p_0}$. $v \in \mathbb{S}^1$ is an arbitrary parameter, and $\vec{n}(v,t)$ is the normal to the closed curve $L(v,t)$. By definition we have $U_{p_0}(L(v,t)) = t$, and by differentiation of this equation by $t$ and $v$ it can be deduced that $U_{p_0}$ satisfies the Eikonal equation

$$||\nabla U_{p_0}|| = \widetilde{P} \quad \text{with} \quad U_{p_0}(p_0) = 0. \tag{5}$$

This equation can be numerically solved using the fast marching method [7] because of its lower complexity compared to the direct front propagation approach implied by (3) while maintaining the same spirit of front propagation in the way that the grid points are visited during the marching procedure.

### 2.2. Dual-front active contours with flexibly global minimizers

In this section, we briefly review the dual-front active contour model [34]. We assume that an image $I$ has two regions $R_{\text{in}}$ and $R_{\text{out}}$ with $B$ as their common boundary. We choose one point $p_0$ from $R_{\text{in}}$ and another point $p_1$ from $R_{\text{out}}$, then we define a velocity $1/\widetilde{P}$ taking lower values near the boundary $B$ and define two minimal action maps $U_{p_0}(p)$ and $U_{p_1}(p)$ according to (2). Contrary to just considering the saddle point $p'$ which satisfies $U_{p_0}(p') = U_{p_1}(p')$ and (3) simultaneously, we consider the set of points $p_e$ which satisfies $U_{p_0}(p_e) = U_{p_1}(p_e)$. These points $p_e$ form a partition curve $B'$ which divides $I$ into two regions. This partition is also a velocity- (or potential-) weighted Voronoi diagram. The region containing $p_0$ will be referred to as $R'_{\text{in}}$, while the other region containing $p_1$ will be referred to as $R'_{\text{out}}$. All points in $R'_{\text{in}}$ are closer (in this weighted sense) to $p_0$ than to $p_1$ and contrariwise for points in $R'_{\text{out}}$. Because the action maps are potential weighted distance maps which can be endowed with Riemannian metrics, $B'$ is called *the potential weighted minimal partition curve*.

The level sets of $U_{p_0}$ and $U_{p_1}$ represent the evolving fronts, and the front velocity $1/\widetilde{P}$ takes on lower values near $B$. When an evolving front arrives at the actual boundary $B$, it evolves slowly and therefore takes a long time to cross $B$. By

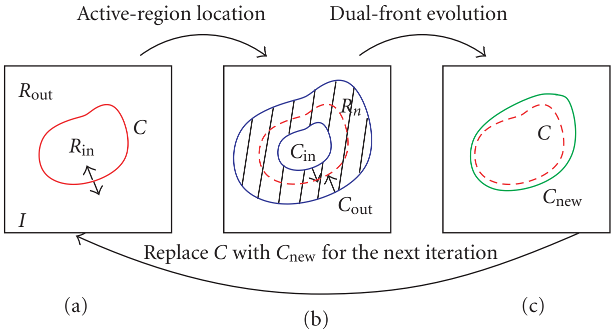

Figure 1: Iteration process of dual-front evolution and active-region location. (a) An initial contour $C$ separates image $I$ to two regions $R_{in}$ and $R_{out}$; (b) the curve $C$ is dilated to form a narrow active region $R_n$; (c) the inner and outer boundaries $C_{in}$ and $C_{out}$ of $R_n$ are set as the initializations of two minimal action maps $U_{C_{in}}$ and $U_{C_{out}}$, and the set of meeting points of the level sets of $U_{C_{in}}$ and $U_{C_{out}}$ forms a new minimal partition curve $C_{new}$ inside $R_n$. $C_{new}$ divides image $I$ into two regions. Curve $C$ is replaced by curve $C_{new}$ for the next iteration.

choosing appropriate potentials when defining $U_{p_0}$ and $U_{p_1}$, we may cause the partition curve $B'$ formed by the meeting points of the level sets of $U_{p_0}$ and $U_{p_1}$ to correspond with the actual boundary $B$. In other words, we can detect $B$ by setting appropriate potentials and finding the minimal partition curve $B'$.

Now let us consider minimal action maps having a set of starting points. Similar to the definitions in [39], we let $X$ be a set of points in image $I$ (e.g., $X$ is a 2D curve or a 3D surface), and define a minimal action map $U_X(p)$ as the minimal energy integrated along a path between a starting point $p_0 \in X$ and any point $p \notin X$:

$$U_X(p) = \min_{p_0 \in X} \left( \inf_{A_{p_0,p}} \left\{ \int_\Omega \widetilde{P}(C(s))\,ds \right\} \right). \quad (6)$$

We choose a set of points $X_i$ from $R_{in}$ and another set of points $X_j$ from $R_{out}$, and define two minimal action maps $U_{X_i}(p)$ and $U_{X_j}(p)$ according to (6). All points satisfying $U_{X_i}(p) = U_{X_j}(p)$ form a partition boundary $B''$ and divide $I$ into two regions. One region contains $X_i$ and the other region contains $X_j$. Because $U_{X_i}(p)$ and $U_{X_j}(p)$ are the potential weighted distance maps, $B''$ is a potential weighted minimal partition of $I$. With appropriate potentials, it is also possible that $B''$ is exactly the actual boundary $B$ of $R_{in}$ and $R_{out}$.

Therefore, the dual front evolution principle proposed in [33] is to find a potential weighted minimum partition curve within an active region.

This principle is shown in Figure 1. A narrow active region $R_n$ is formed by extending an initial curve $C$. For example, it may be generated from $C$ using morphological dilation. $R_n$ has an inner boundary $C_{in}$ and an outer boundary $C_{out}$. Two minimal action maps $U_{C_{in}}$ and $U_{C_{out}}$ are defined by different potentials $\widetilde{P}_{in}$ and $\widetilde{P}_{out}$, respectively, based on (6). When the level sets of $U_{C_{in}}$ and $U_{C_{out}}$ meet each other, the meeting points form a potential weighted minimal partition curve $C_{new}$ in active region $R_n$. The evolution of curves $C_{in}$ and $C_{out}$ and their meeting locations $p_g$ can also be obtained using the "time of arrival" functions which satisfy Eikonal equations

$$\begin{aligned} \|\nabla U_{C_{in}}\| &= \widetilde{P}_{in} \quad \text{with } U_{C_{in}}(C_{in}) = 0, \\ \|\nabla U_{C_{out}}\| &= \widetilde{P}_{out} \quad \text{with } U_{C_{out}}(C_{out}) = 0, \\ U_{C_{in}}(p_e) &= U_{C_{out}}(p_e) \quad \text{on } C_{new}. \end{aligned} \quad (7)$$

Since the dual front evolution is to find the global minimal partition curve only within an active region, not in the whole image, the degree of this global minima changes flexibly by adjusting the size of active regions.

The dual-front active contour model is an iterative process including the dual front evolution followed by relocation of the active region. The minimum partition curve formed by the dual front evolution is extended to form a new active region. We extract the boundaries of the new active region, and define potentials for the evolution of the separated boundaries again. Then we repeat the dual front evolution and the active region location to find new global minimal partition curves until certain stopping conditions are satisfied. For example, we may compare the difference between two consecutive minimal partition curves, to determine when we have converged to a final result.

As shown in (7), two minimal action maps $U_{C_{in}}$ and $U_{C_{out}}$ may be obtained by solving Eikonal equations. In the minimal path technique proposed in [35], they used fast marching methods described in [7] to solve Eikonal equations. Tsitsiklis [42] first used heap-sort structures to solve Eikonal equations, Sethian [7] and Helmsen et al. [43] reported similar approaches lately. Fast marching methods are computationally efficient tools to solve Eikonal equations, in which upwind difference schemes and heap-sort algorithms are used for guaranteeing the solution is strictly increasing or decreasing on grid points. The computational complexity of fast marching methods is $\mathcal{O}(N \log N)$, where $N$ is the number of grid points, and $\log N$ comes from the heap-sort algorithm.

Another algorithm for solving Eikonal equations is the fast sweeping method presented in [37, 44]. It is for computing the solution of Eikonal equations on a rectangular grid based on iteration strategies. In fast sweeping methods [37, 44], the characteristics are divided into a finite number of groups according to their directions and each sweep of Gauss-Seidel iterations with a specific order covers a group of characteristics simultaneously. $2^n$ Gauss-Seidel iterations ($n$ is the spatial dimension) with alternating sweeping order are used to compute a first order accurate numerical solution for the distance function in $n$ dimensions. Fast sweeping methods have an optimal complexity of $\mathcal{O}(N)$ for $N$ grid points, which are extremely simple to implement in any dimension, and give similar results as fast marching methods. The details of fast sweeping methods may be seen in [37, 44].

Both fast marching methods and fast sweeping methods can be used in the dual front evolution for finding the minimal partition curve in an active region. In this paper, the dual front evolution scheme utilizes fast sweeping methods because of its low complexity $\mathcal{O}(N)$, where $N$ is the number

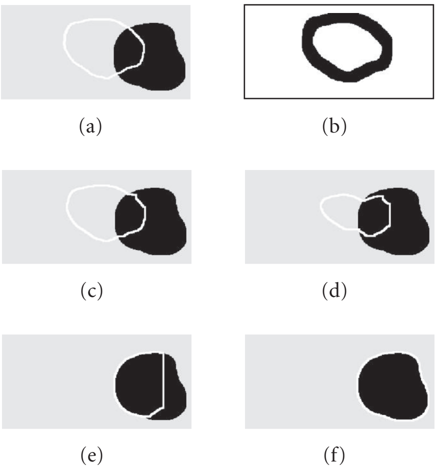

Figure 2: The segmentation result on a 2D synthetic image based on dual-front active contours. (a) The original image and the initial curve (the red line), (b) the black region is the defined active region which is extended from the initial curve using morphological dilation, (c) the new formed global partition curve within the active region after dual-front evolution, (d) to (f) the different new global minimal partition curves after 5, 10, 15 iterations.

of grid points in $R_n$. Because the low computational cost of fast sweeping methods is maintained, and the calculation of all minimal action maps can be finished simultaneously, the complexity of the dual front evolution is still $\mathcal{O}(N)$, where $N$ is the number of grid points in an active region. The 3D dual front evolution scheme is shown in the appendix.

In dual front active contours, potentials may combine region and edge-based information. For example, we may consider the mean values $\mu_{\text{in}}$, $\mu_{\text{out}}$, the variance values $\sigma_{\text{in}}^2$, $\sigma_{\text{out}}^2$ of region $(R_{\text{in}} - R_{\text{in}} \cap R_n)$ and region $(R_{\text{out}} - R_{\text{out}} \cap R_n)$ to decide the evolution potentials for the labeled points $(x, y)$ as

$$\begin{aligned} \widetilde{P}_{\text{in}}(x, y) &= w_{\text{in}}^r f\left(\left|I(x, y) - \mu_{\text{in}}\right|, \sigma_{\text{in}}^2\right), \\ &\quad + w_{\text{in}}^b g\left(\nabla I(x, y)\right) + w_{\text{in}} \quad \text{if } l(x, y) = l_{\text{in}}, \\ \widetilde{P}_{\text{out}}(x, y) &= w_{\text{out}}^r f\left(\left|I(x, y) - \mu_{\text{out}}\right|, \sigma_{\text{out}}^2\right), \\ &\quad + w_{\text{out}}^b g\left(\nabla I(x, y)\right) + w_{\text{out}} \quad \text{if } l(x, y) = l_{\text{out}}, \end{aligned} \tag{8}$$

where $\omega_{\text{in}}^r$ and $\omega_{\text{out}}^r$ are positive weights for the region-based terms, $\omega_{\text{in}}^b$ and $\omega_{\text{out}}^b$ are positive weights for the edge-based items, and $\omega_{\text{in}}$ and $\omega_{\text{out}}$ are positive constants for controlling the smoothness of the partition curves. We choose $g(\nabla I(x, y))$ as a positive decreasing function of the image gradient, and $f$ as a function related to the region-based information. As with any segmentation algorithm, the optimal set of parameters is very application-dependent.

In Figure 2, we give the segmentation process on a 2D synthetic image to show the basic principle of dual-front active contours. Here we use morphological dilation to define the active region for each iteration. The structuring element for morphological dilation was a $15 \times 15$ circle mask. For this example, the potential at a point $(x, y)$ was $\widetilde{P}(x, y) = (|I(x, y) - \mu_l| + 0.1)$, where $\mu_l$ is the mean value of points having the same label $l$ as the point $(x, y)$.

### 2.3. Properties of dual-front active contours

The dual front active contour model has several nice properties. First, the final contour is not just a local minimizer. It possesses a controllable global minima related to certain active regions which vary according to the user-specified amount of dilation used to form the active regions at each step. The result of the dual front evolution is a potential weighted global minimum partition curve within an active region. So the size and shape of active regions affect the final segmentation result. This ability to gracefully move from capturing minima that are more local to minima that are more global makes it much easier to obtain "desirable" minimizers (which often are neither the most local nor the most global).

In Figure 3, we demonstrate that by choosing different active regions with different sizes, dual-front active contours may achieve different global minima within different active regions. Here, the potential at a point $(x, y)$ was defined as $\widetilde{P}(x, y) = |I(x, y) - \mu_l| + (1 + |\nabla I|)^2/10 + 0.1$, where $\mu_l$ is the mean value of points having the same label $l$ as the point $(x, y)$.

Most edge-based active contour models [4, 40] were designed to find local minima of data-dependent energy functionals with the hope that reasonable initial placement of the active contour will drive it towards a "desirable" local minimum rather than an undesirable configuration that can occur due to noise or complex image structure. The minimal path technique proposed by Cohen and Kimmel [35, 38, 39] attempts to capture the global minimum of an active contour model's energy between two user-defined points. Furthermore, a large class of region-based models, such as [45–47], have utilized image information not only near the evolving contour but also image statistics inside and outside the contour in order to improve the performance. Most of these more global region-based energy functionals assume highly constrained models for pixel intensities within each region, and require a priori knowledge of the number of region types. Sometimes, though, minimizers that are too global (or region-based energies using information that is too global) are just as undesirable as minimizers that are too local.

An example of this is illustrated in Figure 4. In this figure, we compare geodesic active contours [40], the minimal path technique [35], Chan-Vese's method [45], and Mumford-Shah method [47] with dual-front active contours. The test image is part of a 2D human brain MRI image, and the objective is to find the interface of gray matter and white matter. The image size is $80 \times 80$ pixels. The structure element for morphological dilation in dual-front active contours is a $5 \times 5$ circle. As this figure indicates, dual-front active contours can control the degree of global or local minima which are related to active regions, find correct boundaries, and perform better than the other methods.

Second, the computational cost of dual-front active contours is reduced significantly. The iteration process in dual front active contours causes the initial and intermediate curves move in large "jumps" in order to arrive at the objective boundary, which substantially reduces the number of

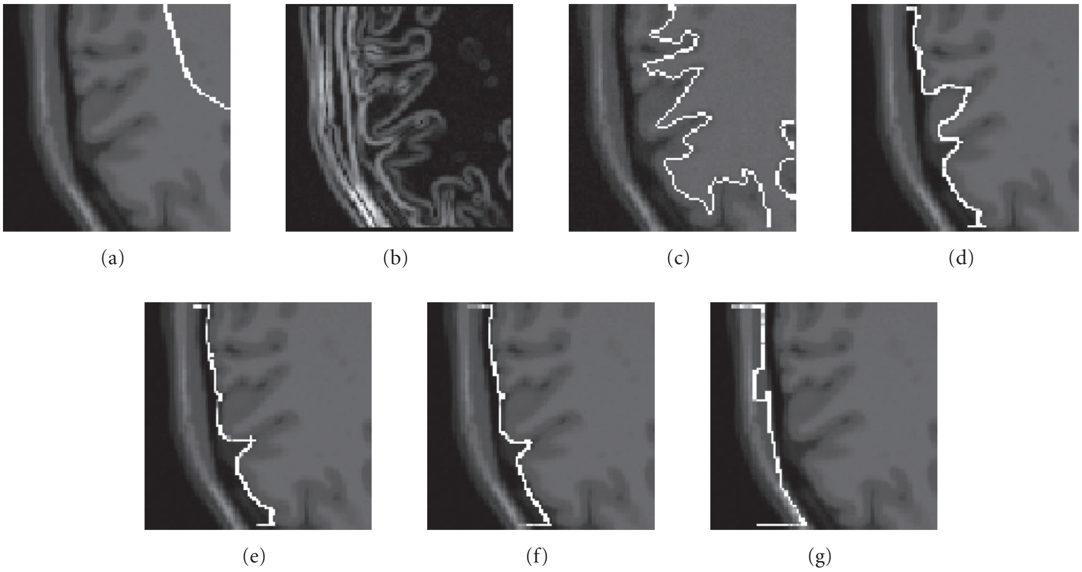

Figure 3: By choosing active regions with different sizes, the dual-front active contour model may achieve different global minima related to different active regions and iteration times. (a) The original image with the initialization. (b) The corresponding gradient information. (c) Segmentation result using a $5 \times 5$ structuring element with 15 iterations of morphological dilation. (d) Segmentation result using a $7 \times 7$ structuring element with 15 iterations of morphological dilation. (e) Segmentation result using an $11 \times 11$ structuring element with 15 iterations of morphological dilation. (f) Segmentation result using a $15 \times 15$ structuring element with 15 iterations of morphological dilation. (g) Segmentation result using a $23 \times 23$ structuring element with 15 iterations of morphological dilation.

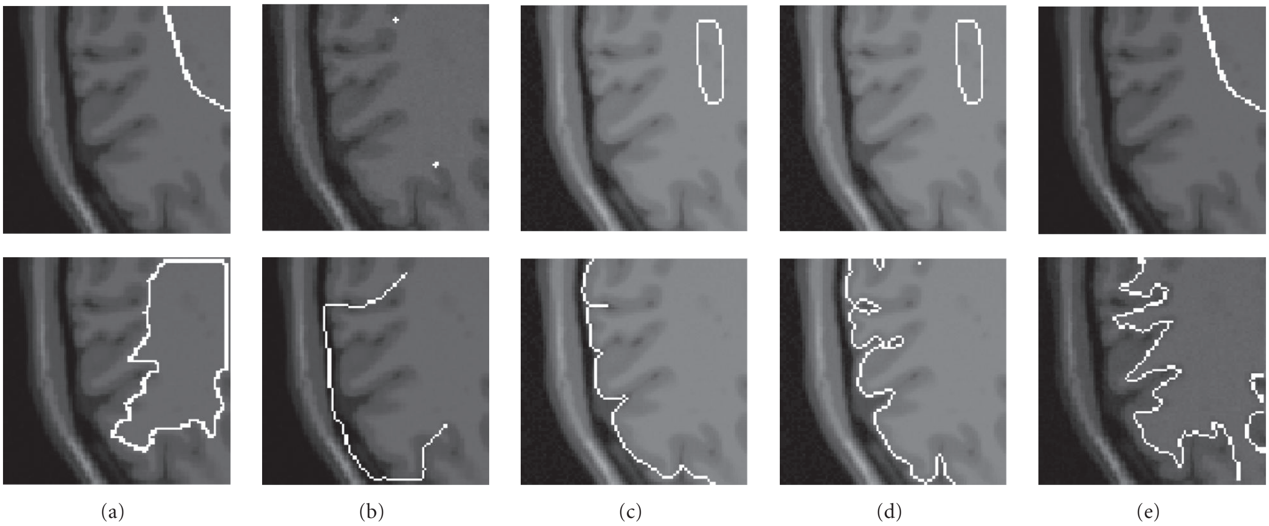

Figure 4: Comparison of different segmentation results for the white matter and gray matter boundaries using different active contour models. The gradient information used in panels (a), (b), and (e) is shown in Figure 3. The top row shows the original image and initializations. The bottom row shows the corresponding segmentation results. (a) Geodesic active contours suffer from undesirable local minima; (b) the minimal path technique relies on the location of initial points and strong gradient information; (c) and (d) Chan-Vese method and Mumford-Shah method find more global minima over the whole image. (e) Improved edge extraction using dual-front active contours with the same initialization used for geodesic active contours.

iterations needed to converge. We also use a fast sweeping numerical scheme [37] for the dual front evolution because of its lower complexity ($\mathcal{O}(N)$, where $N$ is the number of grid points in the band). As a result, the dual-front active contour model enjoys a low complexity of $\mathcal{O}(N)$.

Third, dual-front active contours provide an automatic stopping criterion. In the dual front evolution, whenever two contours from the same group meet, they merge into a single contour. On the other hand, whenever two contours from different groups meet, both contours stop evolving and a common boundary is formed by their meeting points automatically. The iterative process of dual-front active contours stops when the difference between two consecutive minimal partition curves is less than a predefined amount.

Fourth, in dual-front active contours, we provide a very flexible way to define active regions. Generally, we use morphological dilation to generate an active region around the current curve. In this way, the size and shape of the active region can be controlled easily by adjusting the associated structuring elements and dilation times. However, by regarding the active region as a restricted search space, we may use methods such as those presented for active contours with restricted search spaces in [48–51] to form the active regions.

A final observation to make about the dual-front active contour model is to note that while it is vaguely related to a variety of couple surface models [14, 16, 17, 22] discussed in Section 1, it is an altogether different approach. Coupled surfaces models were proposed to evolve a pair of curves together to find a pair of desired contours while exploiting some sensible constraints between the two curves as they evolved. The dual front active contour model, however, seeks to find a single potential weighted minimal partition curve within an active region, which is formed by the meeting points of dual evolving curves. By iteratively forming a new narrow active region based on the current partition curve and then using the dual front evolution to find a new partition curve, dual-front active contours can find the boundary of a *single* desired object. Furthermore, the "dual fronts" can be generalized to multiple fronts. The boundaries of an active region may be composed of multiple separating curves, each independent curve evolves with different potential and different label, whenever two (or more) evolving curves meet each other, both (or more) curve evolutions stop at the meeting points. All the meeting points form a partition curve automatically. The full details are outlined in the appendix.

Table 1: Comparison of tissue segmentation results of our method. Dual-front active contour (1) using morphological dilation to generate the active regions and (2) using histogram analysis to generate active regions.

| Rate | Dual-front active contours (1) | | | Dual-front active contours (2) | | |
|---|---|---|---|---|---|---|
| | CSF | GM | WM | CSF | GM | WM |
| TP (%) | 96.3 | 78.0 | 84.3 | 96.6 | 93.3 | 95.1 |
| FN (%) | 3.7 | 22.0 | 15.7 | 3.4 | 6.7 | 4.9 |
| FP (%) | 44.6 | 13.0 | 5.7 | 5.7 | 5.6 | 5.8 |
| Overlap metric | 0.666 | 0.689 | 0.797 | 0.914 | 0.883 | 0.898 |

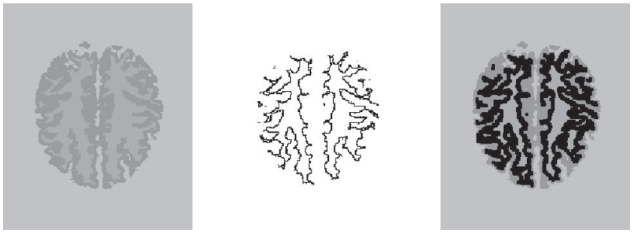

(a) (b) (c)

Figure 5: Morphological dilation affects the topology structure of evolving fronts and also affects the accuracy of segmentation results. (a) The formed partition diagram on one slice after a number of iterations and different regions with different gray values represent different tissues; (b) the extracted boundary between GM and WM tissues on the same slice as that shown in (a); (c) the formed active region (the most black region) by dilating the boundary shown in (b).

### 3. CORTEX SEGMENTATION BY DUAL-FRONT ACTIVE CONTOURS

### 3.1. 3D brain cortex segmentation with flexible user interaction

Due to the complex and convoluted nature of the human brain cortex and partial volume effects of MRI imaging, the brain cortex segmentation must be considered in three dimensions. In this section, we give a 3D brain cortex segmentation scheme based on dual-front active contours.

Generally, in dual front active contours, morphological dilation is used to form an active region from an initial curve. However, it is not a good choice to form active regions for segmentation of the brain cortex. One example is illustrated in Table 1 and the corresponding 3D models are shown in Figure 9. The test image is generated from the normal brain database, BrainWeb [52], using T1 modality, 1 mm slice thickness, 3% noise level, and 20% intensity nonuniformity settings (INU). we assume the brain skull is stripped and that other nonbrain tissues are also removed. The remainder consists of only three kinds of tissues: WM, GM, and CSF. The initialization for this segmentation was a sphere mask centered at $(100, 100, 95)$ with size $75 \times 75 \times 150$. The potential at a point $(x, y, z)$ was chosen as $\widetilde{P}(x, y, z) = (|I(x, y, z) - \mu_l| + 0.1)$, where $\mu_l$ is the mean value of the points having the same label $l$ as the point $(x, y, z)$. The structuring element used for morphological dilation was a $7\times7\times7$ sphere mask. We first used dual-front active contours to segment the CSF boundary, and then processed only the remaining interior region to capture the WM/GM boundary.

As shown in Table 1, the quantitative evaluation of the segmentation result using this morphological dilation is not very good, as the dilation process is blind to the complex and convoluted structure of the brain cortex. Because of the convoluted geometry of the cortex, each time we dilate the partition curve to form a new active region for next iteration, the dilation may change the topology of the partition curve. An example is illustrated in Figure 5. The 3D models shown in panels (a) and (b) of Figure 9 demonstrate the poor performance of this morphological approach.

Since morphological dilation is not the only way to obtain active regions, we may choose another method. Here we propose a scheme based on histogram analysis. This scheme is simple, fast, and accurate with flexible user interaction. In Figure 6, we show an overall diagram of this scheme.

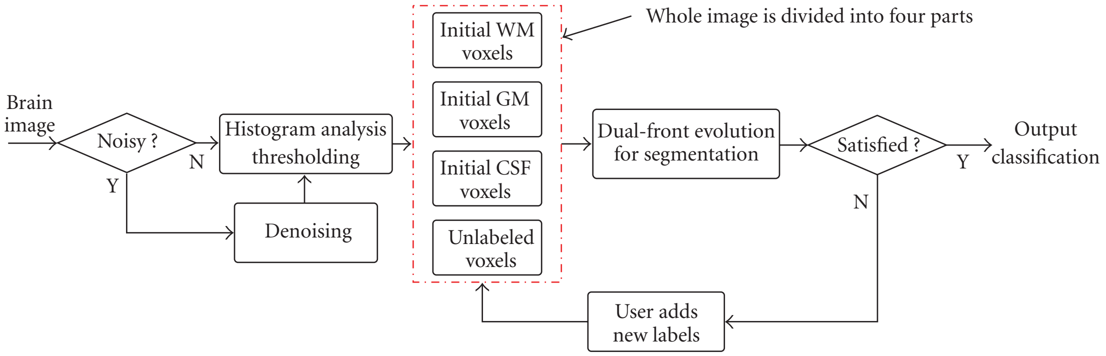

Figure 6: Overall diagram of 3D brain cortex segmentation process.

In this scheme, we first assume the brain skull is stripped and that other nonbrain tissues are also removed. The remainder consists of three kinds of tissues: white matter (WM), gray matter (GM), and cerebrospinal fluid (CSF). If an image is noisy, we first preprocess it to reduce the effects. Next, we divide the whole brain image into four regions: WM seed voxels, GM seed voxels, CSF seed voxels, and unlabeled voxels. All the voxels in the same region (WM, GM, or CSF) have the same label. The background is ignored for the computation. Here, the unlabeled voxels make the "active region" among the labeled WM, GM, CSF voxels, and the active region may be composed of isolated points, sets of points, and so forth. After running the dual front evolution, all the points in the active region are assigned a label which is one of the three labels for GM, WM, and CSF. The final grid is therefore separated into these three tissue classes.

If this initial segmentation does not give satisfactory results, users can modify the initial active region just by adding (or deleting) some labels (via mouse-clicks) as desired, afterwhich the dual-front evolution is automatically rerun to yield an updated segmentation.

### 3.2. Active region and potential decision based on histogram analysis

In this section, we describe a method for creating the active regions, required by the dual-front active contour, by analyzing histograms of normal MRI brain images. A histogram, which is simply a frequency count of the gray levels in the image, is important in many areas of image processing, such as segmentation and thresholding. Analysis of histograms gives useful information about image contrast. For 3D T1-weighted brain cortex images, the reasonable contrast is obtained between the three main tissue classes in brain, which are GM, WM, and CSF. Some brain cortex segmentation approaches [24, 25, 53] were based on automatic gray-level thresholding, and in common, a histogram is first determined, from which the threshold levels are determined by Gaussian fitting algorithms to produce a binary mask. Five Gaussian representing three pure tissue classes (GM, WM, CSF) and two partial volume compartments (GM/WM, CSF/GM) are fitted at a local level and are used to generate either discrete or continuous segmentations [24]. But the problems with these methods include their sensitivity to partial volume effects, which produces speckled regions in the final segmentation. In order to reduce the impact of the noise, some Markov-random-field-based methods [28–31] were proposed.

Figure 7 shows the histogram analysis of these three brain tissues. panel (a) is the histogram of a sample 3D MRI elderly brain image. There are three peaks and two troughs in this histogram. The locations of these peaks approximate the average mean values of the GM, WM, and CSF tissues. The regions around these two troughs correspond to the voxels located around the boundaries of different tissues. Because of the effect from noise and partial volume problems, it is hard to locate the actual boundaries just by simple thresholding.

In this paper, we use histogram analysis for a special purpose. After a histogram is first determined, we may use simple thresholding to choose regions which include the actual boundaries instead of the boundaries themselves. We treat all the voxels in these chosen regions as unlabeled voxels and use a dual-front active contour to assign a unique label to each voxel in this region. The 3D dual front evolution scheme is detailed in the appendix. This process is shown in panel (b) and panel (c) of Figure 7. By setting different thresholds, a 3D brain image may be divided into GM seeds, WM seeds, CSF seeds, and unlabeled voxels which comprise two active regions $R_1$ and $R_2$ around the two troughs in the histogram. As shown in panel (c) of Figure 7, a 3D brain image may be separated into different regions by the previous procedure. The voxels with different gray values represent different initial CSF, GM, and WM voxels. The most black voxels represent the unlabeled pixels which compose the active region. The 3D dual-front evolution scheme may be used to assign a unique label to each voxel in this active region. For images with high noise levels, we smooth the image first and then calculate the histogram. The main purpose of this smoothing process is to eliminate extraneous peaks/troughs in the histogram. Then we may use thresholding to separate an image into different regions. While smoothing makes some parts of the boundaries list distinct, quantitative analysis on several sample images indicate that a limited amount of smoothing actually improves the segmentation results. The details are given in Section 4.1.

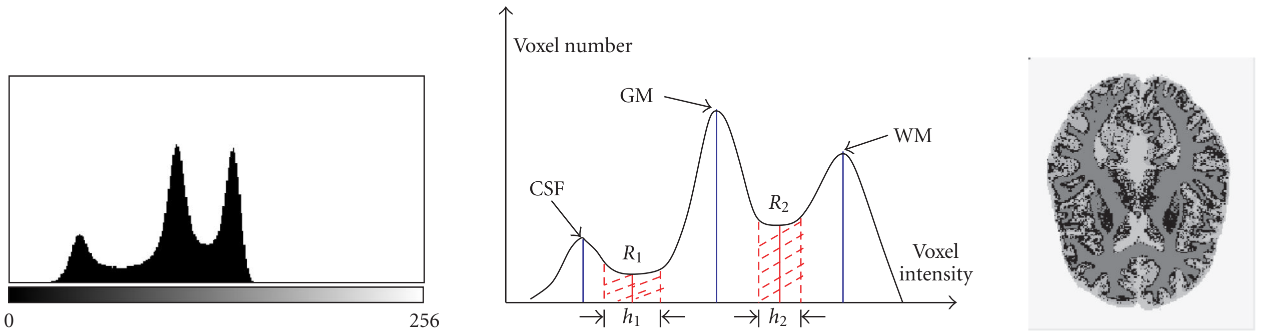

(a) (b) (c)

FIGURE 7: Active regions are determined by histogram analysis and thresholding of 3D MRI brain images. (a) The histogram of a sample 3D MRI brain image; (b) the center of $R_1$ is the trough between the CSF and GM peaks, the center of $R_2$ is the trough between the GM and WM peaks. $h_1$ and $h_2$ decide the size of $R_1$ and $R_2$. (c) The brain image is separated into different regions by thresholding. The most black voxels represent the unlabeled pixels, and voxels with different gray values represent different initial CSF, GM, and WM voxels.

We use region-based information during the front evolutions in our scheme because the tested MRI images rarely provide reliable edge information. We calculate the mean values $\mu_{\text{CSF}}$, $\mu_{\text{GM}}$, and $\mu_{\text{WM}}$, and the variance values $\sigma^2_{\text{CSF}}$, $\sigma^2_{\text{GM}}$, $\sigma^2_{\text{WM}}$, of the three different seed voxel classes with labels $l_{\text{CSF}}$, $l_{\text{GM}}$, and $l_{\text{WM}}$. Then the potential for a labeled point $(x, y, z)$ is set to

$$\widetilde{P}_l(x, y, z) = \omega_1 \cdot \exp\left(\frac{|\bar{I}(x, y, z) - \mu_l|^2}{2\sigma_l^2}\right) + \omega_2 \quad \text{if } L(x, y, z) = l \ (l = l_{\text{CSF}}, l_{\text{GM}}, l_{\text{WM}}), \tag{9}$$

where $\bar{I}(x, y, z)$ is the average image intensity in a window of size $3 \times 3 \times 3$ around the given voxel. $\omega_1$ is a real positive weight for the region-based image potential, while $\omega_2$ is a real positive constant to control the smoothness of the partition curves obtained from the dual front evolution.

### 4. EXPERIMENTAL RESULTS

In this section, we validate our approach on various 3D simulated and real MRI brain image data sets. We use T1-weighted images on account of their better GM/WM contrast [14, 54]. All the experimental results shown in this section are obtained from 3D volume processing directly.

To evaluate the efficiency of our method for every tissue type $T$ (GM, WM, and CSF), four probabilities are defined:

$$\text{TP} = \frac{N_B \cap N_R}{N_R}, \qquad \text{FN} = \frac{N_R - N_B \cap N_R}{N_R}, \qquad \text{FP} = \frac{N_B - N_B \cap N_R}{N_R}, \qquad \text{OM} = \frac{\text{TP}}{1 + \text{FP}}, \tag{10}$$

where $N_R$ is the number of reference ground truth voxels of tissue $T$. $N_B$ is the number of voxels detected by our algorithm for tissue $T$. $N_B \cap N_R$ is the number of correct voxels detected by our method for tissue $T$. TP means true positive, which is the probability of correct detection relative to the ground truth for tissue $T$. FN means false negative, which is the probability of misdetection relative to the ground truth for tissue $T$. FP means false positive, which is the probability of false detection relative to the ground truth for tissue $T$. OM means overlap metric, which is defined for a given voxel class assignment as the sum of the number of voxels that both have the class assignment in each segmentation divided by the sum of voxels where either segmentation has the class assignment [55]. This measurement is more critical than comparisons using the volume only, it is the same as the Tanimoto coefficient [56]. This metric approaches a value of 1.0 for results that are very similar and is near 0.0 when they share no similarly classified voxels. In the following experiments we use these parameters to quantitatively analyze our segmentation results.

### 4.1. *Validation on simulated MR brain images*

In Figure 8, we present the results of the segmented WM tissues for five different slices of one 3D simulated brain image provided by BrainWeb [52], which is generated from the normal brain database using the T1 modality, 1 mm slice thickness, 3% noise level, and 20% intensity nonuniformity settings (INU).

For this segmentation, we use the potentials defined by (9) with $\omega_1 = 1$ and $\omega_2 = 0.1$. The size of $R_1$, $h_1$ is equal to 20, and the size of $R_2$, $h_2$ is equal to 10 (shown in Figure 7). In fact, $\omega_1$ and $\omega_2$ are two parameters for adjusting potentials, while $h_1$ and $h_2$ are two parameters for adjusting the size of active regions. The best or most appropriate values for these parameters have to be chosen for different classes of images. In this paper, we manually choose these parameters by testing on a few sample images, and then using the same values for all of the rest. Adaptive tuning of these parameters is one of the subjects for future research.

In Table 1, we give the quantitative results of two brain cortex segmentations on the same 3D simulated image as that in Figure 8. This 3D simulated brain image is provided by BrainWeb [52], and is generated from the normal brain database using the T1 modality, 1 mm slice thickness, 3%

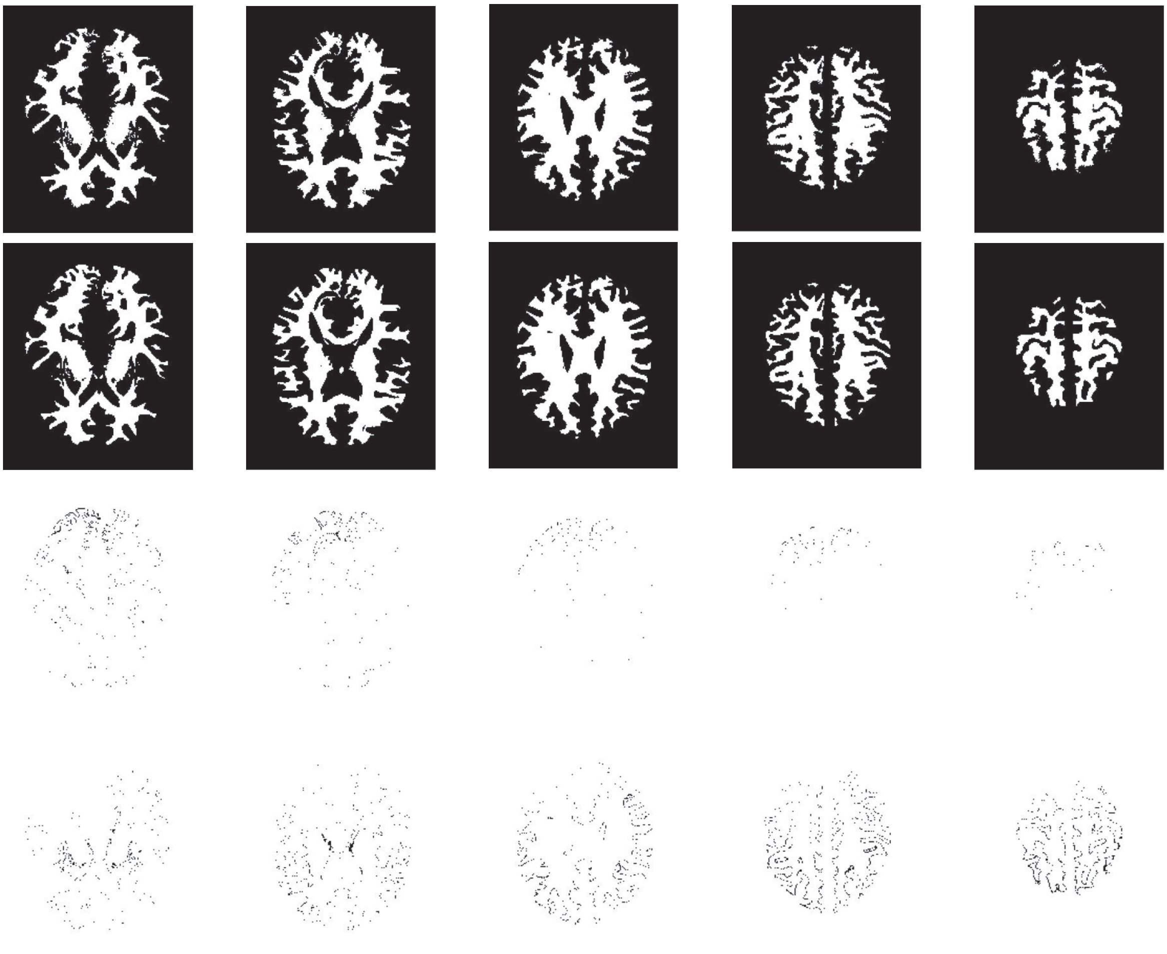

(a) (b) (c) (d) (e)

Figure 8: Comparison of the segmentation results from our method with the ground truth data of five slices of one 3D simulated brain image provided by BrainWeb [52], which is of T1 modality, 1 mm slice thickness, 3% noise level, 20% INU. The image size is $181 \times 217 \times 181$. The top row presents the segmentation results obtained from our method. The second row shows the ground truth data provided by BrainWeb database. The third row shows the false negative difference between the segmentation results from our method and the ground truth data. The fourth row shows the false positive difference between the segmentation results from our method and the ground truth data. These five columns correspond to five slices of the test 3D image.

noise level, and 20% intensity nonuniformity settings (INU). One result is obtained by using dual front active contours with active regions obtained by morphological dilation. The second result is obtained by using dual front active contours with active regions obtained by the same histogram analysis as in Figure 8. Figure 9 shows the corresponding 3D models of the segmented GM and WM surfaces from these two methods explained in Table 1 and from the ground truth data. From these segmentation results we can see that our scheme based on histogram analysis performs better than the dual-front active contours based on morphological dilation.

We also tested our method on 15 3D simulated brain images provided by BrainWeb [52, 57], which are of T1 modality, 1 mm slice thickness, different noise levels 1%, 3%, 5%, 7%, and 9%, and different INU settings 0%, 20%, and 40%. All images are $181\times217\times181$. For this segmentation, we continued to use the same potentials defined by (9) with $\omega_1 = 1$ and $\omega_2 = 0.1$. The size of $R_1$, $h_1$ is 20, and the size of $R_2$, $h_2$ is 10.

For images with high noise levels 5%, 7%, and 9%, we first use the isotropic nonlinear diffusion proposed by Perona and Malik [58] to smooth the images. Since the ground truth data is also provided by BrainWeb website, it is easy for us to compare the accuracy from the original images and the corresponding smoothed image. For the segmented results, the overlap metrics of three tissues for 5 images with different noise levels and same 0% INU setting are from 0.813 to 0.944, the overlap metrics of three tissues for 5 images with different noise levels and same 20% INU setting are from 0.814 to 0.914, the overlap metrics of three tissues for 5 images with different noise levels and same 40% INU setting are from 0.747 to 0.835. In Figure 10, we also show these segmentation results. In Figure 10, some CSF segmentation results of images having 0% INU are worse than the results of images

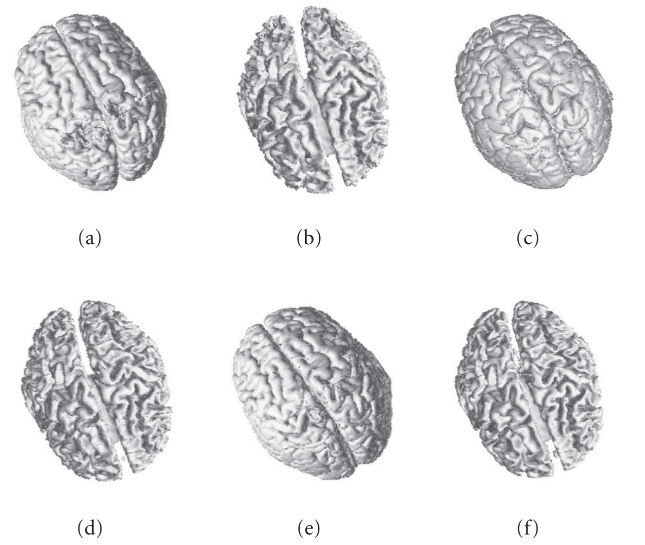

Figure 9: Comparison of the 3D models of GM and WM surfaces from our method and from the ground truth data. The test image is the same as in Figure 8. (a) and (b) are the 3D models of the GM and WM surfaces obtained from our method using morphological dilation; (c) and (d) are the 3D models of the GM and WM surfaces obtained from our method using histogram analysis; (e) and (f) are the 3D models of the GM and WM surfaces from the ground truth.

having 20% INU. We think there are three reasons. One is that the two parameters $h_1$ and $h_2$ are constants. The chosen parameters are not the best or most appropriate values for processing all the images and segmenting all the tissues. Furthermore, when we choose these parameters, we consider more on their performance on the GM and WM segmentation results than that of the CSF segmentation results. The best or most appropriate values for these parameters have to be chosen based on different applications. How to set adaptive tuning of these parameters is very application-dependent and still needs further research work. The second reason is that we use some smoothing operators to smooth the images with noise levels 5%, 7%, and 9% first and then segment them. These smoothing processes may also effect the final results. Furthermore, CSF tissues are much thinner than GM and WM tissues, which also may effect the segmentation results.

### 4.2. Validation on real MR brain images

To further evaluate our segmentation method under more realistic conditions, we test it on 20 real MRI brain images and compare the segmentation results with those of human experts as well as to those obtained by other segmentation algorithms. These 20 normal MR brain data sets are provided by the Center for Morphometric Analysis at Massachusetts General Hospital on the IBSR website http://www.cma.mgh.harvard.edu/ibsr/. The IBSR website also provides the segmentation results on GM, WM, and CSF tissues from the adaptive MAP method, the biased MAP method, the fuzzy $C$-means method, the maximum a posteriori probability method, the maximum-likelihood method, and the tree-structure $k$-means method on these 20 normal brain-only MR data sets along with the manual segmentation results on GM and WM tissues from two experts [59]. Since the segmentation results provided by the IBSR website are measured by two parameters "overlap metric" and "average overlap metric," we will also measure the results from our method by these same two parameters for the sake of meaningful comparison.

Figure 11 shows the overlap metric of CSF, GM, and WM segmentation results (compared to expert manual results) on 20 normal brains for various automatic segmentation results provided by IBSR, for the hidden Markov method [28] provided by the FMRIB website (http://www.fmrib.ox.ac.uk/fsl/), and for our proposed scheme. For the segmentation of these real brain images, we still use the potentials defined by (9) with $\omega_1 = 1$ and $\omega_2 = 0.1$. The size of $R_1$, $h_1$ is 20, the size of $R_2$, $h_2$ is 10.

Figure 12 shows the average overlap metric of GM and WM segmentation results on these 20 normal brains provided by the IBSR website for various methods. The figures show that the overlap metric and the average overlap metric of the segmentation results from our method are either higher than or at least close to the other methods. However, the computational time for our method is around 20 seconds, which is much faster than other methods.

In these comparisons shown in Figures 11 and 12, in addition to the comparison with the methods provided by IBSR [55, 60], we also compare our method with other three recently proposed methods, the Bayesian method proposed in [27] (MPM-MAP); the coupled surfaces method [14] (ZENG), and the hidden Markov method [28] (FAST). This study is just the initial step of our research work on brain image analysis. We will still work on it, and try to improve the model's robustness and the segmentation's accuracy further.

### 4.3. Simple and useful user interaction

In the previous two subsections, various segmentation results of our scheme on simulated and real 3D brain datasets are shown, and simple thresholding operators are used for defining active regions in the dual-front evolution. Generally, most automatic techniques, while less demanding on the user, are much less accurate. It would be useful to employ a fast automatic segmentation procedure to do most of the work but still allow an expert user to interactively guide the segmentation to ensure an accurate final result. An attractive feature of our scheme is that it is extremely simple for users to add seed points just by mouse clicks to yield corrections to the segmentation that extend far beyond their initial locations (due to the flexibly global nature of dual front active surfaces), thus minimizing the user effort. Figure 13 shows an example of this interaction.

In Figure 13, we use the same image as the one used for the test shown in Figure 8. One slice of the test 3D image (panel (a)), the ground truth data for the WM tissue in this slice (panel (b)), and the 3D model of the ground truth WM tissue (panel (c)) are shown in the first row. The second row

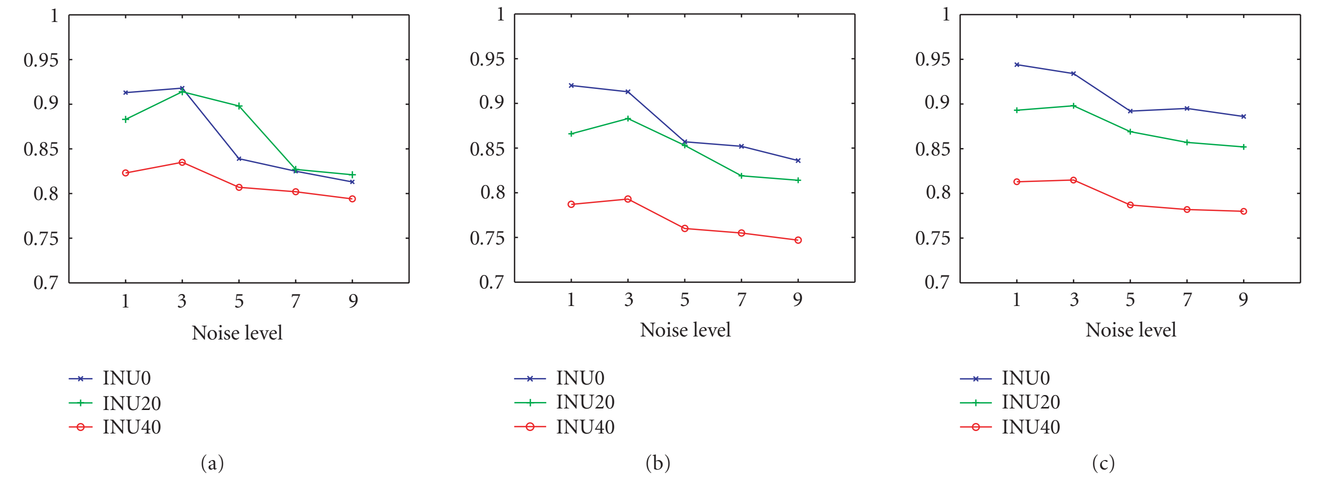

FIGURE 10: Overlap metric for CSF, GM, and WM segmentations on simulated brain images provided by BrainWeb website. The different noise levels are 1%, 3%, 5%, 7%, and 9%. The three curves labeled INU0, INU20, and INU40 represent the overlap metric of the segmentation results of the images with 0%, 20%, and 40% INU settings based on our proposed method. (a) The overlap metric of CSF segmentation results for images with different noise levels (1%, 3%, 5%, 7%, and 9%) and different INU settings (0%, 20%, and 40%). (b) The overlap metric of GM segmentation results for images with different noise levels (1%, 3%, 5%, 7%, and 9%) and different INU settings (0%, 20%, and 40%). (c) The overlap metric of WM segmentation results for images with different noise levels (1%, 3%, 5%, 7%, and 9%) and different INU settings (0%, 20%, and 40%).

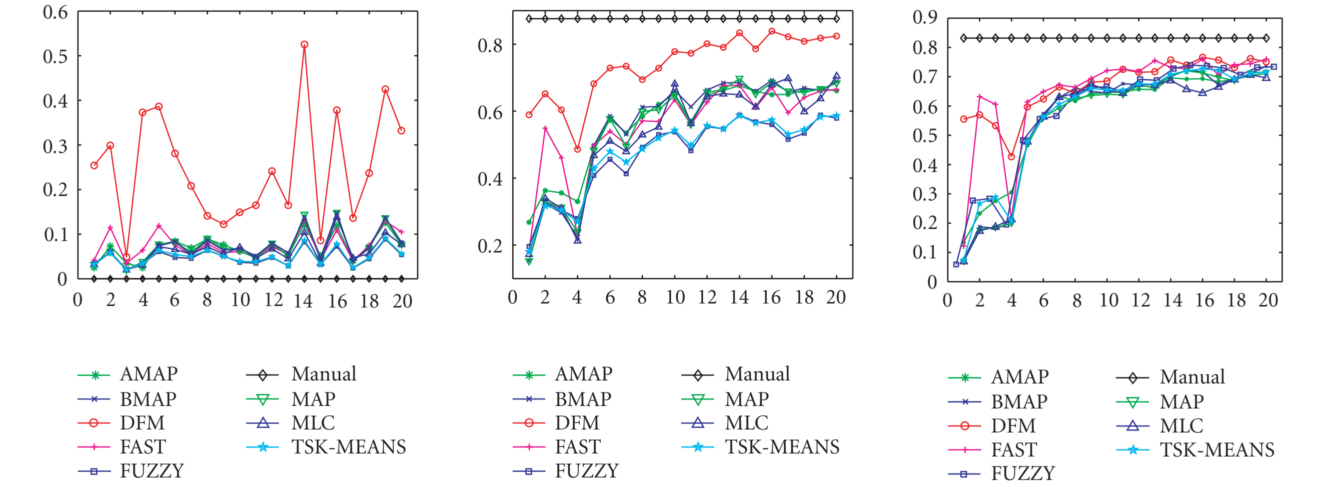

(a) Overlap metric comparison for CSF segmentation

(b) Overlap metric comparison for GM segmentation

(c) Overlap metric comparison for WM segmentation

FIGURE 11: The overlap metric of CSF, GM, and WM segmentations results on 20 normal real brain images for various segmentation methods. The results of some automatic segmentation methods provided by IBSR. AMAP: adaptive MAP; BMAP: biased MAP; FUZZY: fuzzy *C*-means; MAP: maximum a posteriori probability; MLC: maximum likelihood; TSKMEANS: tree-structure *k*-means; FAST: hidden Markov method [28]; DFM: our scheme.

shows the segmentation result using dual-front active contours, in which active regions are chosen based on automatic thresholding. In this test, we set a different active region between the WM and GM tissues by changing the size and location of $R_2$ according to Figure 7. The most black region in panel (d) presents unlabeled voxels in the active region, and different regions with different gray values represent different tissues' initial seed points. panel (e) shows the segmentation result. The 3D model of the segmented WM is shown in panel (g). These figures illustrate that if automatic thresholding cannot provide enough WM seed points, the segmented WM tissue may be incorrect. So in addition to employing a fast automatic segmentation procedure to do most of the work, it would be useful to still allow an expert user to interactively guide the segmentation to ensure an accurate final result. We show segmentation result after user interaction in the third row of Figure 13. As shown in panel (h), the user interaction simply consists of a few mouse clicks to add

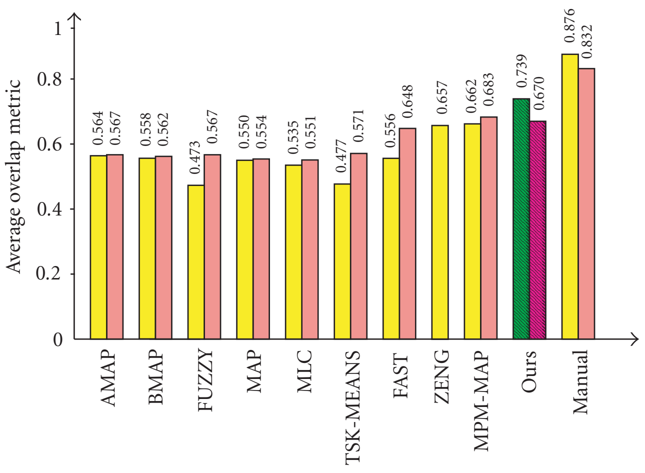

Figure 12: Average overlap metric for GM and WM segmentations on 20 normal real brains for various segmentation methods. The results of some automatic segmentation methods were provided by IBSR or related papers. For each method, the left column represents the average overlap metric of GM segmentation, the right column represents the average overlap metric of WM segmentation. For our method, the left column represents the average overlap metric of GM segmentation, the right column represents the average overlap metric of WM segmentation. AMAP: adaptive MAP; BMAP: biased MAP; FUZZY: fuzzy $C$-means; MAP: maximum a posteriori probability; MLC: maximum likelihood; TSK-MEANS: tree-structure $k$-means; FAST: hidden Markov method [28]; ZENG: coupled-surface method [14]; MPM-MAP: Bayesian method [27]; DFM: our scheme.

some new seed points. We then run the dual-front evolution again to segment the GM and WM. The segmented boundary of GM/WM is shown in panel (i), the extracted WM tissue and the corresponding 3D model are shown in panel (j) and panel (k). The figures show that the accuracy of the result after user interaction is much better than that just based on automatic thresholding.

We provide a flexible way to combine histogram analysis and dual-front active contours. We may first set certain predefined parameters such as the different weights in potentials, and the width of $h_1$ and $h_2$, then do histogram analysis and the dual front evolution to obtain the segmentation result directly. We may also do histogram analysis separately and let experts choose appropriate parameters for the dual-front evolution based on histogram analysis and their experience. In most fully automatic methods, users need to repeat the whole process to obtain different results, and it is hard to tune the associated parameters flexibly. But our method provides a fast automatic segmentation procedure to do most of the work but still allow an expert user to interactively guide the segmentation to ensure an accurate final result.

### 4.4. *Computational time*

Another nice property of our method is its high computational efficiency. We test our method on 15 simulated 3D MR brain images provided by BrainWeb [57], and 20 real normal 3D MR brain images provided by IBSR website. The average computational time is around 20 seconds on a 2.5 GHz Pentium4 PC processor, out of which the average computational time for the histogram analysis is about 5 seconds and the average computational time for the dual front evolution is less than 15 seconds.

Since most methods introduced in Section 1 were tested on different images and run on the different processors, it is hard for us to give exact quantitative comparisons on the computational time between our method and these other methods. Here we just give a brief discussion on the computational time reported for various cortex segmentation methods.

We downloaded the software for the hidden Markovian method from the website of the FMRIB Software Library (http://www.fmrib.ox.ac.uk/fsl/) to compare its computational speed with our method. On the same computer, the average computational time for the hidden Markovian method for same test images was around 550 seconds. Xu's method [13] combined the adaptive fuzzy $C$-means algorithm [15]; they reported that the computational time for the final deformable surface algorithm was about 3 hours using an SGI workstation with a 174 MHz R10000 processor.

For the coupled surface method proposed by Zeng et al. [14], it was reported that for a 3D image of the whole brain with a voxel size of $1.2 \times 1.2 \times 1.2\,\text{mm}^3$, their algorithm runs in about 1 hour on a SGI Inigo2 machine with a 195 MHz R10000 processor for the implementation of skull stripping, cortex segmentation, and measurement simultaneously. Goldenberg et al. [17] also adopted the coupled surfaces principle and used the fast geodesic active contour approach to improve the computational time for cortex segmentation. They reported that the computational time of their method was about 2.5 minutes for a $192 \times 250 \times 170$ MR image of the whole brain on a Pentium3 PC. But they did not give the quantitative analysis of the segmentation results.

Teo et al. [11] reported that their entire procedure takes about 0.5 hours. Prior to the procedure, gray matter needs to be identified manually in a single occipital lobe of one hemisphere using rudimentary segmentation tools, which requires about 18 hours for an expert. Much of the time is spent on visually inspecting connectivity and ensuring topological correctness. In MacDonald's method [16], the processing time for each object was reported to be 30 hours on an SGI Origin 200 R10000 processor running at 180 MHz. Dale et al. [12] reported that their entire procedure runs automatically in about 1.5 hours. Kapur et al. [10] reported that their method required about 20 minutes to process a single 3D image.

In Marroquin's method [27], it was reported that the average total processing time (including registration for peeling the skull and nonbrain material and segmentation) on 20 normal brain data sets from IBSR is 29 minutes on a single processor of an SGI ONYX machine. In the adaptive fuzzy C-means algorithm (AFCM) [15], they reported that execution times for 3D T1-weighted MR data sets with 1 mm cubic voxels are typically between 45 minutes and 3 hours when using full multigrid AFCM, and that execution

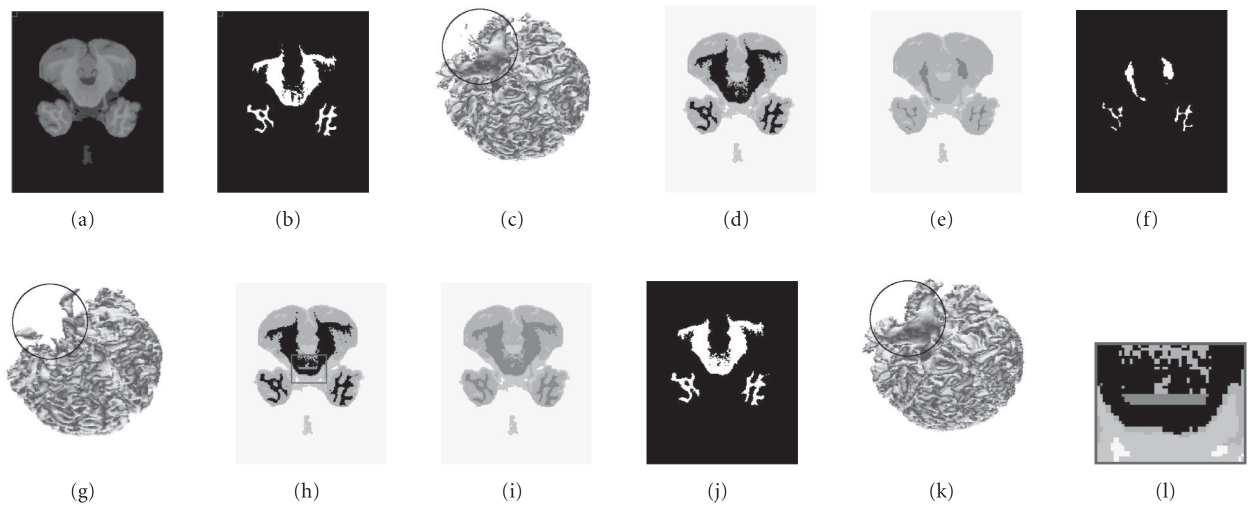

FIGURE 13: Simple user interaction can improve the segmentation accuracy dramatically (see text). (a) One slice of the original 3D brain image, (b) the ground truth data of WM tissue in the slice shown in panel (a), (c) the corresponding 3D model of ground truth data of WM tissue, (d) based on histogram analysis, the whole brain is divided into GM tissue, WM tissue, CSF tissue with different gray values, and unlabeled tissues in active regions with the most black values, (e) the segmentation result by using dual-front active contours, (f) the segmented WM tissue (the white part), (g) the 3D model of segmented WM tissue, (h) manually added seed points for WM tissue, (i) the new segmentation result with added seed points, (j) the segmented WM tissue, (k) the corresponding 3D model of the segmented WM tissue, (l) the horizontal line is the zoom-in of the user-added seed points in panel (h).

times are between 10 minutes and 1 hours when using truncated multigrid AFCM. In the graph-based topology correction algorithm (GTCA) [18] proposed by Han et al., they reported that the processing time depends on the total number of foreground/background filters required. For the brain volumes with typical size $140 \times 200 \times 160$ used in their experiments, each filter took less than 3 minutes on an SGI Onyx2 workstation with a 250 MHz R10000 processor, and the total processing time for each brain volume took less than 10 minutes. Normally, manual segmentation of one type tissue segmentation for an experienced person is about 18 hours.

### 5. CONCLUSIONS AND FUTURE WORK

In this paper, we proposed a novel scheme for 3D brain cortex segmentation based on dual-front active contours and local histogram analysis. The experimental section illustrated several advantages of our scheme. The first is that our scheme exhibited better results than most other methods when tested on 20 real normal brain images as demonstrated in Figures 11 and 12. The second is that the average computational time of our method is less than 20 seconds, which is much faster than most other methods, as discussed in Section 4.4. The third is that our method facilitates optional user interaction which is crucial when highly accurate results are needed, as it allows a trusted and trained user to guide the segmentation processes. This is discussed and illustrated in Section 4.3.

Our future research work will continue on the following aspects because of the complexity and variety of medical brain images. Since the dual-front active contour model is fast and easy to implement, it is easily combined with other preprocessing and postprocessing methods to improve the segmentation accuracy further. From the segmentation results shown in Section 4, we can see, for images with high INU settings and high noise levels, the segmentation results are not as good. We will work on combing our current model with INU bias compensation methods and smoothing methods to improve its performance in these conditions. In recent years, several methods have been proposed to correct INU settings [15, 26], and some other methods were also proposed to remove image noise. We will investigate on how other methods might be used in conjunction with our model.

Second, we have just used potentials based on region-based information because the interfaces between different tissues in the tested images were not very clear (due to partial volume effects). However, edge-based information is important and widely exploited for image segmentation and feature extraction. We are working on developing more robust local edge operators, and combining them with region-based information in our potentials to further improve the accuracy of our results.

Third, our model can be generalized to multispectral data sets commonly used in MR imaging. When processing such data sets, vectors may be used to represent intensities of image voxels instead of scalars, and how to design appropriate potentials and active regions for this case is a very interesting topic needing further investigation.

Fourth, we use histogram analysis to determine the active regions. The test images in this paper are normal elderly brain images, but young normal histograms have small CSF compartments compared to those seen in the elderly histograms. Additionally, in diseased brains, the contrast between gray and white matter is considerably reduced, and the two histogram peaks sometimes merge. In fact, when we tested our method on two of the 20 real MR brain images, we had to choose the cutoff values for the active regions manually. Now, we are working on finding better methods to choose active regions for improving the method's generality.

### APPENDIX

### 3D DUAL-FRONT EVOLUTION SCHEME

#### *Initialization*

Label map $L$: Initial contours $B_1, \dots, B_n$ with labels $l_i, \dots, l_n$; otherwise, $l(p) = -1$.

Action map $U$: for any point $p$ of the initial contours, set $U(p) = 0$; for other points, set $U(p) = \infty$.

Potentials $\widetilde{P}_{l_i}(p)$: which is calculated based on the label $l_i$ of the point $p$.

*Input*: active region $R_n$ in image $A$ $(I \times J \times K)$, initial label map $L$, initial action map $U$.

#### *Sweeping forward loop*

(1) For each point $x(i, j, k)$ in $R_n$, calculate its new label and new action value by the ordering $i = 1 \to I$, $j = 1 \to J$, $k = 1 \to K$, as the following steps:

(i) the new label of $x$ is the label of the point, which has the smallest $U$ value among the point $x$ and its 6-connected neighbors,

$$\begin{aligned} x_{\min} = \{x \mid u(x) = \\ &\min\big(u_{i,j,k}, u_{i-1,j,k}, u_{i+1,j,k}, u_{i,j-1,k}, \\ &\quad u_{i,j+1,k}, u_{i,j,k-1}, u_{i,j,k+1}\big)\}; \\ l^{\text{new}}_{i,j,k} = l(x_{\min}); \end{aligned} \tag{A.1}$$

(ii) calculate the new potential $h^{\text{new}}_{i,j,k}$ of point $x$, and find three minimum $U$ in the 6-connected neighbors of point $x$:

$$\begin{gathered} h^{\text{new}}_{i,j,k} = \widetilde{P}_{l^{\text{new}}_{i,j,k}}(i, j, k); \\ a = \min\big(u_{i-1,j,k}, u_{i+1,j,k}\big); \qquad b = \min\big(u_{i,j-1,k}, u_{i,j+1,k}\big); \\ c = \min\big(u_{i,j,k-1}, u_{i,j,k+1}\big); \end{gathered} \tag{A.2}$$

(iii) arrange $a$, $b$, and $c$ as $U_A \le U_B \le U_C$, and calculating new $U$ from the current value of its 6-connected neighbors:

(a) if $|U_A - U_B| \ge h^{\text{new}}_{i,j,k}$, $\overline{u_{i,j,k}} = U_A + h^{\text{new}}_{i,j,k}$,
(b) if $|U_A - U_B| < h^{\text{new}}_{i,j,k}$, $\Delta_1 = 2(h^{\text{new}}_{i,j,k})^2 - (U_B - U_A)^2$,
(c) if $\sqrt{\Delta_1} \le 2U_C - (U_A + U_B)$, $\overline{u_{i,j,k}} = (U_A + U_B) + \sqrt{\Delta_1}/2$,
(d) if $\sqrt{\Delta_1} > 2U_C - (U_A + U_B)$, $\Delta_2 = (U_A + U_B + U_C)^2 - 3(U_A^2 + U_B^2 + U_C^2 - (h^{\text{new}}_{i,j,k})^2)$,

$$\overline{u}_{i,j,k} = (U_A + U_B + U_C) + \frac{\sqrt{\Delta_2}}{3}, \tag{A.3}$$

– updating $u_{i,j,k}$: $u^{\text{new}}_{i,j,k} = \min(u_{i,j,k}, \overline{u})$.

(2) Repeat the above computation $2^3$ times with alternating sweeping orders.

*Output* is the label map $L$ which divides the active region $R_n$ to $n$ regions.

### ACKNOWLEDGMENTS

The authors would like to thank the anonymous reviewers for valuable comments and suggestions, and to thank Dr. Sebastien Fourey and Dr. Regis Clouard of GREYC-ENSICAEN, France, for providing MVox 3D model visualization and PANDORE image processing platform. This work is supported by NSF Grant CCR-0133736 and NIH Grant R01NS037747.

### REFERENCES

[1] M. Kass, A. Witkin, and D. Terzopoulos, "Snakes: active contour models," *International Journal of Computer Vision*, vol. 1, no. 4, pp. 321–332, 1988.
[2] L. D. Cohen, "On active contour models and balloons," *Computer Vision Graphics Image Processing: Image Understanding*, vol. 53, no. 2, pp. 211–218, 1991.
[3] L. D. Cohen and I. Cohen, "Finite-element methods for active contour models and balloons for 2D and 3D images," *IEEE Tranactions on Pattern Analysis and Machine Intelligence*, vol. 15, no. 11, pp. 1131–1147, 1993.
[4] A. Yezzi, S. Kichenassamy, A. Kumar, P. Olver, and A. Tannenbaum, "Geometric snake model for segmentation of medical imagery," *IEEE Transactions on Medical Imaging*, vol. 16, no. 2, pp. 199–209, 1997.
[5] R. Malladi, R. Kimmel, D. Adalsteinsson, G. Sapiro, V. Caselles, and J. A. Sethian, "Geometric approach to segmentation and analysis of 3D medical images," in *Proceedings of the Workshop on Mathematical Methods in Biomedical Image Analysis (MMBIA '96)*, pp. 244–252, San Francisco, Calif, USA, June 1996.
[6] R. Malladi and J. A. Sethian, "A real-time algorithm for medical shape recovery," in *Proceedings of the IEEE International Conference on Computer Vision*, pp. 304–310, Bombay, India, January 1998.
[7] J. A. Sethian, "A fast marching level set method for monotonically advancing fronts," *Proceedings of the National Academy of Sciences of the United States of America*, vol. 93, no. 4, pp. 1591–1595, 1996.
[8] D. Adalsteinsson and J. A. Sethian, "A fast level set method for propagating interfaces," *Journal of Computational Physics*, vol. 118, no. 2, pp. 269–277, 1995.
[9] C. Davatzikos and R. N. Bryan, "Using a deformable surface model to obtain a shape representation of the cortex," *IEEE Transactions on Medical Imaging*, vol. 15, no. 6, pp. 785–795, 1996.
[10] T. Kapur, W. E. L. Grimson, W. M. Wells III, and R. Kikinis, "Segmentation of brain tissue from magnetic resonance images," *Medical Image Analysis*, vol. 1, no. 2, pp. 109–127, 1996.
[11] P. C. Teo, G. Sapiro, and B. A. Wandell, "Creating connected representations of cortical gray matter for functional MRI visualization," *IEEE Transactions on Medical Imaging*, vol. 16, no. 6, pp. 852–863, 1997.
[12] A. M. Dale, B. Fischl, and M. I. Sereno, "Cortical surface-based analysis," *NeuroImage*, vol. 9, no. 2, pp. 179–194, 1999.
[13] C. Xu, D. L. Pham, M. E. Rettmann, D. N. Yu, and J. L. Prince, "Reconstruction of the human cerebral cortex from magnetic resonance images," *IEEE Transactions on Medical Imaging*, vol. 18, no. 6, pp. 467–480, 1999.

[14] X. Zeng, L. H. Staib, R. T. Schultz, and J. S. Duncan, "Segmentation and measurement of the cortex from 3D MR images using coupled-surfaces propagation," *IEEE Transactions on Medical Imaging*, vol. 18, no. 10, pp. 927–937, 1999.

[15] D. L. Pham and J. L. Prince, "Adaptive fuzzy segmentation of magnetic resonance images," *IEEE Transactions on Medical Imaging*, vol. 18, no. 9, pp. 737–752, 1999.

[16] D. MacDonald, N. Kabani, D. Avis, and A. Evans, "Automated 3D extraction of inner and outer surfaces of cerebral cortex from MRI," *NeuroImage*, vol. 12, no. 3, pp. 340–356, 2000.

[17] R. Goldenberg, R. Kimmel, E. Rivlin, and M. Rudzsky, "Cortex segmentation: a fast variational geometric approach," *IEEE Transactions on Medical Imaging*, vol. 21, no. 12, pp. 1544–1551, 2002.

[18] X. Han, C. Xu, U. Braga-Neto, and J. L. Prince, "Topology correction in brain cortex segmentation using a multiscale, graph-based algorithm," *IEEE Transactions on Medical Imaging*, vol. 21, no. 2, pp. 109–121, 2002.

[19] R. Valdés-Cristerna, V. Medina-Bañuelos, and O. Yáñez-Suárez, "Coupling of radial-basis network and active contour model for multispectral brain MRI segmentation," *IEEE Transactions on Biomedical Engineering*, vol. 51, no. 3, pp. 459–470, 2004.

[20] J. C. Bezdek, *Pattern Recognition with Fuzzy Objective Function Algorithms*, Plenum, New York, NY, USA, 1981.

[21] C. Xu and J. L. Prince, "Snakes, shapes, and gradient vector flow," *IEEE Transactions on Image Processing*, vol. 7, no. 3, pp. 359–369, 1998.

[22] J. Gomes and O. Faugeras, "Reconciling distance functions and level sets," *Journal of Visual Communication and Image Representation*, vol. 11, no. 2, pp. 209–223, 2000.

[23] R. Goldenberg, R. Kimmel, E. Rivlin, and M. Rudzsky, "Fast geodesic active contours," *IEEE Transactions on Image Processing*, vol. 10, no. 10, pp. 1467–1475, 2001.

[24] N. Kovacevic, N. J. Lobaugh, M. J. Bronskill, B. Levine, A. Feinstein, and S. E. Black, "A robust method for extraction and automatic segmentation of brain images," *NeuroImage*, vol. 17, no. 3, pp. 1087–1100, 2002.

[25] Z. Y. Shan, G. H. Yue, and J. Z. Liu, "Automated histogram-based brain segmentation in T1-weighted three-dimensional magnetic resonance head images," *NeuroImage*, vol. 17, no. 3, pp. 1587–1598, 2002.

[26] A. W.-C. Liew and H. Yan, "An adaptive spatial fuzzy clustering algorithm for 3D MR image segmentation," *IEEE Transactions on Medical Imaging*, vol. 22, no. 9, pp. 1063–1075, 2003.

[27] J. L. Marroquin, B. C. Vemuri, S. Botello, F. Calderon, and A. Fernandez-Bouzas, "An accurate and efficient Bayesian method for automatic segmentation of brain MRI," *IEEE Transactions on Medical Imaging*, vol. 21, no. 8, pp. 934–945, 2002.

[28] Y. Zhang, M. Brady, and S. Smith, "Segmentation of brain MR images through a hidden Markov random field model and the expectation-maximization algorithm," *IEEE Transactions on Medical Imaging*, vol. 20, no. 1, pp. 45–57, 2001.

[29] S. Ruan, C. Jaggi, J. Xue, J. Fadili, and D. Bloyet, "Brain tissue classification of magnetic resonance images using partial volume modeling," *IEEE Transactions on Medical Imaging*, vol. 19, no. 12, pp. 1179–1187, 2000.

[30] S. Ruan, B. Moretti, J. Fadili, and D. Bloyet, "Fuzzy Markovian segmentation in application of magnetic resonance images," *Computer Vision and Image Understanding*, vol. 85, no. 1, pp. 54–69, 2002.

[31] K. Van Leemput, F. Maes, D. Vandermeulen, and P. Suetens, "Automated model-based tissue classification of MR images of the brain," *IEEE Transactions on Medical Imaging*, vol. 18, no. 10, pp. 897–908, 1999.

[32] K. M. Pohl, W. M. Wells III, A. Guimond, et al., "Incorporating non-rigid registration into expectation maximization algorithm to segment MR images," in *Proceedings of the 5th International Conference on Medical Image Computing and Computer Assisted Intervention (MICCAI '02)*, pp. 564–572, Tokyo, Japan, September 2002.

[33] H. Li, A. Elmoataz, J. Fadili, and S. Ruan, "Dual front evolution model and its application in medical imaging," in *Proceedings of the 7th International Conference on Medical Image Computing and Computer-Assisted Intervention (MICCAI '04)*, vol. 3216 of *Lecture Notes in Computer Science*, pp. 103–110, Saint-Malo, France, September 2004.

[34] H. Li and A. Yezzi, "Local or global minima: flexible dual front active contours," in *Proceedings of Workshop-Computer Vision for Biomedical Image Applications (CVBIA '05)*, pp. 356–366, Beijing, China, October 2005.

[35] L. D. Cohen and R. Kimmel, "Global minimum for active contour models: a minimal path approach," in *Proceedings of the IEEE Computer Society Conference on Computer Vision and Pattern Recognition (CVPR '96)*, pp. 666–673, San Francisco, Calif, USA, June 1996.

[36] E. Rouy and A. Tourin, "A viscosity solutions approach to shape-from-shading," *SIAM Journal on Numerical Analysis*, vol. 29, no. 3, pp. 867–884, 1992.

[37] H. Zhao, "A fast sweeping method for Eikonal equations," *Mathematics of Computation*, vol. 74, no. 250, pp. 603–627, 2005.

[38] L. D. Cohen and R. Kimmel, "Global minimum for active contour models: a minimal path approach," *International Journal of Computer Vision*, vol. 24, no. 1, pp. 57–78, 1997.

[39] L. D. Cohen, "Multiple contour finding and perceptual grouping using minimal paths," *Journal of Mathematical Imaging and Vision*, vol. 14, no. 3, pp. 225–236, 2001.

[40] V. Caselles, R. Kimmel, and G. Sapiro, "Geodesic active contours," *International Journal of Computer Vision*, vol. 22, no. 1, pp. 61–79, 1997.

[41] T. Deschamps and L. D. Cohen, "Fast extraction of minimal paths in 3D images and applications to virtual endoscopy," *Medical Image Analysis*, vol. 5, no. 4, pp. 281–299, 2001.

[42] J. N. Tsitsiklis, "Efficient algorithms for globally optimal trajectories," *IEEE Transactions on Automatic Control*, vol. 40, no. 9, pp. 1528–1538, 1995.

[43] J. Helmsen, E. G. Puckett, P. Colella, and M. Dorr, "Two new methods for simulating photolithography development in 3D," in *Optical Microlithography IX*, vol. 2726 of *Proceedings of SPIE*, pp. 253–261, 1996.

[44] M. Boué and P. Dupuis, "Markov chain approximations for deterministic control problems with affine dynamics and quadratic cost in the control," *SIAM Journal on Numerical Analysis*, vol. 36, no. 3, pp. 667–695, 1999.

[45] T. F. Chan and L. A. Vese, "Active contours without edges," *IEEE Transactions on Image Processing*, vol. 10, no. 2, pp. 266–277, 2001.

[46] A. Yezzi, A. Tsai, and A. Willsky, "A fully global approach to image segmentation via coupled curve evolution equations," *Journal of Visual Communication and Image Representation*, vol. 13, no. 1-2, pp. 195–216, 2002.

[47] D. Mumford and J. Shah, "Optimal approximation by piecewise smooth functions and associated variational problems,"

*Communications on Pure and Applied Mathematics*, vol. 42, pp. 577–685, 1989.

[48] M. Dawood, X. Jiang, and K. P. Schäfers, "Reliable dual-band based contour detection: a double dynamic programming approach," in *Proceedings of International Conference on Image Analysis and Recognition (ICIAR '04)*, vol. 3212 of *Lecture Notes in Computer Science*, pp. 544–551, Porto, Portugal, September-October 2004.

[49] G. B. Aboutanos, J. Nikanne, N. Watkins, and B. M. Dawant, "Model creation and deformation for the automatic segmentation of the brain in MR images," *IEEE Transactions on Biomedical Engineering*, vol. 46, no. 11, pp. 1346–1356, 1999.

[50] C. E. Erdem, A. M. Tekalp, and B. Sankur, "Video object tracking with feedback of performance measures," *IEEE Transactions on Circuits and Systems for Video Technology*, vol. 13, no. 4, pp. 310–324, 2003.

[51] N. Xu, R. Bansal, and N. Ahuja, "Object segmentation using graph cuts based active contours," in *Proceedings of the IEEE Computer Society Conference on Computer Vision and Pattern Recognition (CVPR '03)*, vol. 2, pp. 46–53, Madison, Wis, USA, June 2003.

[52] BrainWeb, "Mcconnell Brain Imaging Center, Montreal Neurological Institute," http://www.bic.mni.mcgill.ca/brainweb/.

[53] T. J. Grabowski, R. J. Frank, N. R. Szumski, C. K. Brown, and H. Damasio, "Validation of partial tissue segmentation of single-channel magnetic resonance images of the brain," *NeuroImage*, vol. 12, no. 6, pp. 640–656, 2000.

[54] R. T. Schultz and A. Chakraborty, "Magnetic resonance image analysis," in *Handbook of Human Brain Function: Neuroimaging*, E. Bigler, Ed., pp. 9–51, Plenum, New York, NY, USA, 1996.

[55] D. N. Kennedy, P. A. Filipek, and V. S. Caviness Jr., "Anatomic segmentation and volumetric calculations in nuclear magnetic resonance imaging," *IEEE Transactions on Medical Imaging*, vol. 8, no. 1, pp. 1–7, 1989.

[56] R. Duda and P. Hart, *Pattern Classification and Scene Analysis*, John Wiley and Sons, New York, NY, USA, 1973.

[57] C. A. Cocosco, V. Kollokian, R. K.-S. Kwan, and A. C. Evans, "Brain web: online interface to a 3D MRI simulated brain database," *NeuroImage*, vol. 5, no. 4, part 2/4, p. S425, 1997.

[58] P. Perona and J. Malik, "Scale-space and edge detection using anisotropic diffusion," *IEEE Transactions on Pattern Analysis and Machine Intelligence*, vol. 12, no. 7, pp. 629–639, 1990.

[59] J. C. Rajapakse and F. Kruggel, "Segmentation of MR images with intensity inhomogeneities," *Image and Vision Computing*, vol. 16, no. 3, pp. 165–180, 1998.

[60] P. A. Filipek, C. Richelme, D. N. Kennedy, and V. S. Caviness Jr., "The young adult human brain: an MRI-based morphometric analysis," *Cerebral Cortex*, vol. 4, no. 4, pp. 344–360, 1994.

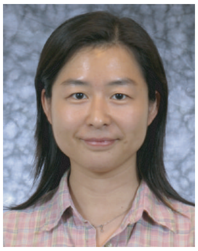

**Hua Li** received her Ph.D. degree in 2001 from the Department of Electronics and Information Engineering at Huazhong University of Science and Technology, China. Currently, she is a Postdoctoral Research Fellow at the School of Electrical and Computer Engineering, Georgia Institute of Technology. Her research interests are in the fields of image processing and computer vision; in particular PDEs, active contour models, curve and surface evolution theory, and mathematical morphology for 3D image segmentation and analysis with applications to medical imaging.

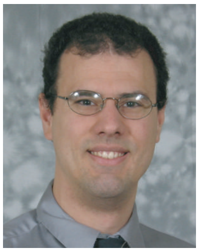

**Anthony Yezzi** obtained his Ph.D. degree in 1997 from the Department of Electrical Engineering at the University of Minnesota. After completing a postdoctoral research position in the Laboratory for Information and Decision Systems (LIDS) at Massachusetts Institute of Technology, he joined the faculty of the School of Electrical and Computer Engineering at Georgia Institute of Technology in 1999, where he currently holds the position of Associate Professor. He has also consulted a number of medical imaging companies including GE, Picker, and VTI, and has been an IEEE Member since 1999. His research lies primarily within the fields of image processing and computer vision. He has worked on a variety of problems including image denoising, edge detection, segmentation and grouping, shape analysis, multiframe stereo reconstruction, tracking, and registration. Some central themes of his research include curve and surface evolution theory, differential geometry, and partial differential equations.

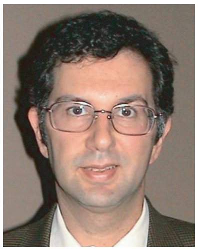

**Laurent D. Cohen** was at École Normale Supérieure, Ulm, in Paris from 1981 to 1985. He received M.S. and Ph.D. degrees in applied mathematics from the University of Paris 6 in 1983 and 1986. From 1985 to 1987, he was a Member at the Computer Graphics and Image Processing Group at Schlumberger Palo Alto Research, California, and Schlumberger Montrouge Research, and remained Consultant there for a few years afterwards. He began working with INRIA, France, in 1988, mainly with the Medical Image Understanding Group Epidaure. Since 1990, he has been a Research Scholar (Charge then Directeur de Recherche) with CNRS in the Applied Mathematics and Image Processing Group at CEREMADE, University Paris-Dauphine. His research interests and teaching at the university are applications of variational methods and partial differential equations to image processing and computer vision, like deformable models, minimal paths, surface reconstruction, image registration, image segmentation and restoration. He obtained CS 2002 Prize for Image and Signal Processing. He has been a member in program committees for boards for about 20 international conferences.

